# Supplementary material for: Photoluminescence enhancement by deterministically site-controlled, vertically stacked SiGe quantum dots
Source: Sci Rep. 2021 Oct 18;11:20597. doi: 10.1038/s41598-021-99966-7 (PMC8523567; doi:10.1038/s41598-021-99966-7)
Supplement: Supplementary file 1 — Supplementary Information. [file 41598_2021_99966_MOESM1_ESM.pdf]

Supplementary Information

A Spatially resolved PL measurements

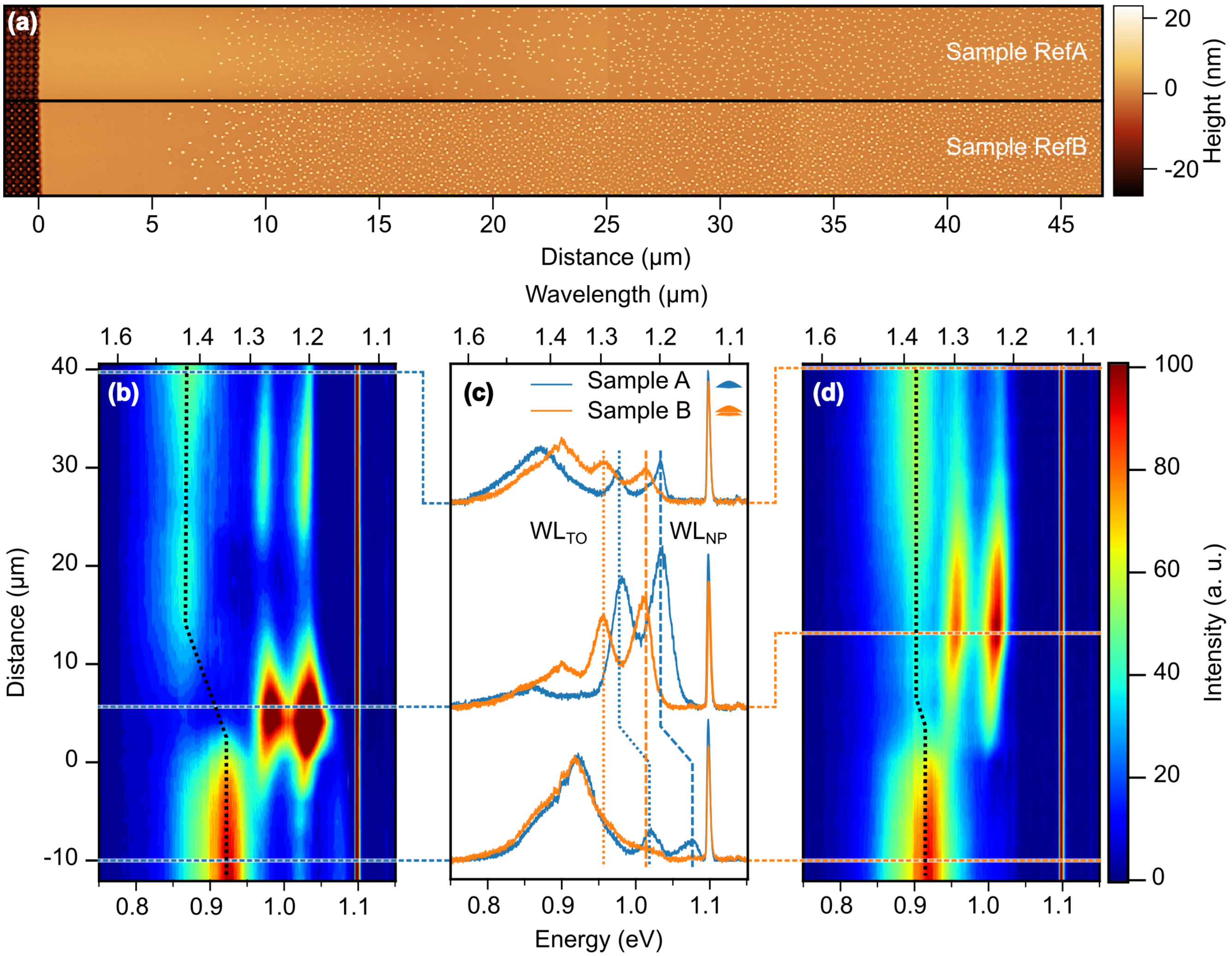

Figure S1. (a) AFM images of the uncapped samples RefA and RefB in the boundary regions between pit-patterned (340 nm period, 100 nm radius) and plain substrate areas. The onset of the pit-pattern defines the origin of the distance scale, with positive values pointing into the plain substrate area. In both samples, a denuded zone free of QDs is observed in the first 5 µm to 7 µm of the plain area, followed by random QD nucleation with a bimodal shape distribution of pyramid- and dome-shaped QDs. (b), (d): Photoluminescence contour plots of sample A and B, respectively, on the same distance scale as in (a). As a guide to the eye, the dotted vertical lines indicate the respective shifts of the QD signals from the patterned to the plain areas. (c) Comparison of PL spectra of the two samples from the ordered field, the denuded zone and the randomly nucleated area (bottom to top), as indicated by the horizontal dashed lines in (b), (d). The dashed vertical lines indicate the energetic shifts of the respective WL-signal peaks. All spectra were recorded at 10 K. This Figure is identical to Fig. 6 in the main manuscript.

Figure S1(a) shows AFM micrographs recorded over a range of 50 µm in the vicinity of a pit-patterned field with 340 nm pit period on the two reference samples RefA and RefB, which lack the Si cap but are otherwise identical to samples A and B (Table 1 in the main manuscript). The distance scale points into the plain region of the substrate, with the origin defined at the boundary to the patterned area. In agreement with Refs.[1,2], we observe a depleted zone free of QDs that covers the first 5 µm to 7 µm into the plain region on both samples. Beyond the depleted zone, randomly nucleated QDs appear. In sample RefA the

QD-density oscillates with increasing distance from the patterned area[2], whereas the stacked-QD sample RefB shows a more homogeneous QD distribution[1].

In Fig. S1 the low-temperature PL spectrum of sample B lacks an apparent WL signal, even though separate WLs for each QD layer in the stack are clearly observed in the cross-sectional STEM images (Fig. 2 in the main manuscript). To gain further insight into this finding, we recorded PL spectra for samples A and B in the sample regions adjacent to the ordered QD arrays. On the plain Si substrate outside the patterned fields, the QDs of the first QD layer nucleate randomly. In the random-nucleation area, the second QD layer is also vertically stacked above the first one (Fig. 2 in the main manuscript) due to the tensile in-plane strain forming locally in the spacer layer[1]. Also, lateral diffusion into the pre-patterned region leads to modulations of the QD densities and of the WL-related PL signal in the plain substrate regions adjacent to the patterned areas[2]. Here, we investigate how the PL signal of a QD stack differs from that of a single QD layer in the boundary region between patterned and un-patterned substrate areas.

Contour plots of the corresponding PL signals are depicted in Figs. S1(b), (d) for samples A and B, respectively. The contour plots are constructed from PL spectra that were recorded every 2 µm on the same distance scale as in Fig. S1(a). The abscissae in Fig. S1(b), (d) show the energy of the PL signal, and the normalized intensity is color-coded from 0 (blue) to 100 % (red). In addition, three pairs of spectra from the two samples are shown in Fig. S1(c) from the patterned, denuded and randomly-nucleated regions, respectively, as indicated by the horizontal dashed lines in Fig. S1(b), (d).

The PL signals of sample A (Fig. S1(b), blue spectra in Fig. S1(c)) behave essentially as described in Ref.[2], i.e., we observe a shift of the PL signal to lower energies and a substantial decrease in intensity on the way into the plain region. The QD signal (black dashed lines in Fig. S1(b), (d)) and the WL signals (WL_{NP} and WL_{TO} indicated by vertical blue and orange dashed lines in Fig. S1(c)) experience a significant blue-shift of 50 meV and 45 meV, respectively, from the plain to the patterned substrate regions. This behavior is well known and was ascribed to a reduced Ge concentration of QDs nucleating in pits caused by enhanced lateral diffusion of Si from the pit walls into the QDs during nucleation in pits[2–4]. The WL between the pits in sample A may also be affected by alloying, however, the observed blue-shift is more likely dominated by a reduced WL thickness in these areas: According to Ref.[5], the etch pits assume the shape of inverted, $\{1\ 1\ 10\}$-facetted pyramids after Si buffer layer growth. During the early stages of Ge deposition, the original pit shape is rotated by 45° into energetically more favorable, inverted pyramids with Ge-terminated $\{1\ 0\ 5\}$-facets and additional $\{1\ 0\ 5\}$-facetted ripple structures (Ref.[5] and inset of Fig. 1(d) in the main manuscript). The shape transformation of the pits requires substantial amounts of Ge which have to diffuse from the WL into the pits before QDs can nucleate there. As a consequence, the WL between the pits should be significantly thinner than in the plain substrate regions, where this kind of sink for Ge-ad-atoms does not exist. The holes in a thinner WL become quantum-confinement-shifted to higher energies which leads to a blue-shift of the associated PL signal. Using the experimentally determined shift of approximately 65 meV per monolayer (ML) WL thickness[6], we estimate that the initial shape transformation of the pits requires the transfer of about 2/3 of a Ge ML from the WL.

The behavior of double-QD sample B is quite different (Fig. S1(d) and orange spectra in Fig. S1(c)). The blue-shift of the QD-related signal from the randomly nucleated to the patterned region is only about 20 meV, i.e., much smaller than for sample A. The nextnano++ simulations showed that only the upper dot, which appears to be less severely alloyed, contributes to the PL signal of the double-QD sample. Outside the patterned area, sample B shows the PL signals (WL_{NP}, WL_{TO}, orange vertical lines in Fig. S1(c)) of a single WL. This finding is consistent with earlier PL investigations of QD stacks with varying spacer layer thicknesses in Ref.[3], where separate WL PL signals for each QD layer could only be observed for spacer layer thicknesses larger than 10 nm. It was assumed in Ref.[3] that for sufficiently thin spacer layers, hole tunneling into the energetically favorable WL suppresses the PL signal from the other one. Comparing the WL signals in the plain substrate region (upper spectra in Fig. S1(c)), we find indeed a red-shift of sample B by 25 meV with respect to sample A.

We assume that the relatively high thermal budget during the growth of the second QD layer is not only responsible for the aforementioned shape and size transformation of the lower QD, but also affects the WLs. In this picture, the WL associated with the lower QDs would transfer Ge to the upper WL during the second Ge-deposition step. Because of the reduced quantum confinement, the observed WL signal from the plain region of sample B should therefore originate from the upper WL. Relating again the observed WL-signal shift to the respective thickness[6], we estimate that the upper WL in sample B is approximately 0.4 ML thicker than the WL of sample A.

As mentioned before, it is surprising that there is no apparent WL signal from the ordered region on sample B. However, when following the WL signal of this sample from the plain substrate area into the ordered region, as indicated by the extended vertical orange lines in Fig. S1(c), a pair of weak shoulders on the high-energy side of the QD signal becomes discernible. This finding is indicative of a weak WL signal in the ordered region of sample B, which stays at the same energy (within 10 meV) as in the plain substrate area. Evidently, the upper WL is decoupled from the aforementioned material loss of the lower WL into the pits. Moreover, the WL signal in the ordered region of sample B is much weaker than in the random region and significantly weaker than the WL signal in the ordered regions of sample A. Evidently, the detrimental radiative recombination channel via the WL is efficiently suppressed in closely stacked, ordered QDs.

B Excitation intensity-dependent PL experiments

In a comparative study of samples A and B, the excitation intensity (I_E) was varied from 3.4 kW cm^{-2} to 1100 kW cm^{-2}. Here, a 532 nm frequency-doubled Nd:YAG laser was employed with the same setup used for the spatially-resolved measurements described above. Figure S2 shows the resulting spectra of the two samples recorded at 70 K. While the shapes of the spectra are quite similar, the integral intensity of the signal from the double-layer QDs is, at all applied I_E, higher than for the single layer. With increasing excitation strength, the intensity of both signals increases. No saturation on either of the samples is reached in the investigated excitation range. The QD PL signal of the single-layer sample (blue) has its maximum at an energy of 0.929 eV for the lowest I_E of 3.4 kW cm^{-2}. Increasing the excitation results in a blue-shift of the peak's central wavelength by 34 meV up to 0.963 eV for the highest I_E of 1100 kW cm^{-2}. On the double-layer sample (orange), the behavior is similar. The central energy shifts from 0.92 eV up to 0.96 eV. The observed blue-shift is indicative of band filling upon increasing excitation. At the highest excitation, the PL intensity of the double-layer sample is about 30 % higher than for the single-layer sample. In each of the spectra, at 1.1 eV, the silicon FE peak is visible, which is broadened at the elevated measurement temperature of 70 K used here. In the inset of Fig. S2, the QD-PL (I_{PL}) integrated between 0.8 eV and 1.07 eV versus I_E is shown for samples A and B in a double-logarithmic plot. The results for both samples can be fitted over the investigated excitation range to a power law $I_{PL} \propto I_E^m$ using the same exponent of $m = 0.7$. Exponents m between 0.6 and 0.7 are typical for PL from SiGe-QDs and have been associated with non-radiative Auger recombination being the dominating mechanism in this material system at the relatively high excitation intensities employed[7].

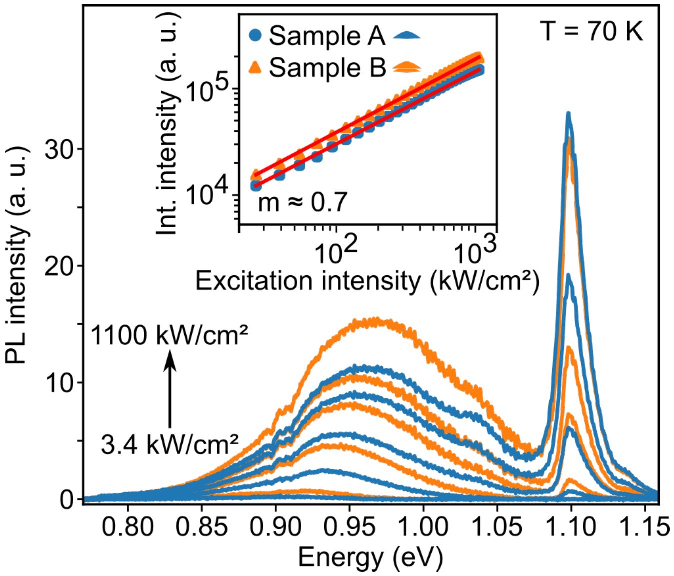

Figure S2. Excitation intensity-dependent μ-PL measurements of samples A (blue) and B (orange) at $T = 70$ K. Measurements are shown for the QD arrays with a period of 340 nm and pit radii of 100 nm. The excitation intensity was varied from 3.4 kW cm^{-2} to 1100 kW cm^{-2}. Increased excitation yields an increased photoluminescence signal intensity and a concurrent shift of the peak's central wavelength towards higher energies from 0.929 eV up to 0.963 eV (sample A) and from 0.920 eV up to 0.960 eV (sample B) for the highest applied excitation. The PL intensity of the double-layer sample exceeds the one of the single-layer sample by approximately 30 %. The peak at 1.098 eV is the temperature-broadened $FE_{TO/LO}$ signal. In the inset the integrated intensities of the QD signals are plotted double-logarithmically as a function of the excitation intensity. Clear exponential behavior with single exponent of $m = 0.7$ is found for both samples.

References

1. Kar, G. S., Kiravittaya, S., Stoffel, M. & Schmidt, O. G. Material Distribution across the Interface of Random and Ordered Island Arrays. *Phys. Rev. Lett.* **93**, 246103 (2004).
2. Grydlik, M. *et al.* Unrolling the evolution kinetics of ordered SiGe islands via Ge surface diffusion. *Phys. Rev. B* **88**, 115311 (2013).
3. Schmidt, O. G. & Eberl, K. Multiple layers of self-asssembled Ge/Si islands: Photoluminescence, strain fields, material interdiffusion, and island formation. *Physical Review B* **61**, 13721 (2000).
4. Zhang, J. J. *et al.* Strain engineering in Si via closely stacked, site-controlled SiGe islands. *Applied Physics Letters* **96**, 193101 (2010).
5. Chen, G., Lichtenberger, H., Bauer, G., Jantsch, W. & Schäffler, F. Initial stage of the two-dimensional to three-dimensional transition of a strained SiGe layer on a pit-patterned Si(001) template. *Phys. Rev. B* **74**, 035302 (2006).
6. Brehm, M. *et al.* Quantitative determination of Ge profiles across SiGe wetting layers on Si (001). *Appl. Phys. Lett.* **93**, 121901 (2008).
7. Tsybeskov, L. & Lockwood, D. J. Silicon-Germanium Nanostructures for Light Emitters and On-Chip Optical Interconnects. *Proc. IEEE* **97**, 1284–1303 (2009).
